# Supplementary material for: Race- and Gender-Specific Associations between Neighborhood-Level Socioeconomic Status and Body Mass Index: Evidence from the Southern Community Cohort Study
Source: Int J Environ Res Public Health. 2023 Nov 30;20(23):7122. doi: 10.3390/ijerph20237122 (PMC10706233; doi:10.3390/ijerph20237122)
Supplement: Supplementary file 1 [file ijerph-20-07122-s001.zip › ijerph-2676382-supplementary.pdf]

Supplemental Tables.

Table S1. Associations between Obese Status and Neighborhood Deprivation, stratified by sex and race, among SCCS at baseline interview, 2002-2009												
	White						Black					
	Males			Females			Males			Females		
Deprivation Index [a]	N, "Healthy" weight [b]	N, Obese [c]	OR (95% CI) [d]	N, "Healthy" weight [b]	N, Obese [c]	OR (95% CI) [d]	N, "Healthy" weight [b]	N, Obese [c]	OR (95% CI) [d]	N, "Healthy" weight [b]	N, Obese [c]	OR (95% CI) [d]
Q1	465	453	1.0 (ref)	738	720	1.0 (ref)	235	300	1.0 (ref)	212	695	1.0 (ref)
Q2	501	759	1.54 (1.28, 1.85)	883	1624	1.55 (1.34, 1.78)	425	486	0.91 (0.72, 1.15)	383	1338	1.04 (0.85, 1.27)
Q3	488	722	1.45 (1.20, 1.76)	807	1706	1.71 (1.48, 1.98)	579	645	1.05 (0.84, 1.31)	381	1787	1.43 (1.18, 1.74)
Q4	559	781	1.39 (1.15, 1.68)	812	1845	1.76 (1.52, 2.03)	1188	1246	1.00 (0.81, 1.23)	844	3911	1.42 (1.19, 1.69)
Q5	576	514	1.03 (0.84, 1.25)	498	1039	1.64 (1.39, 1.93)	5282	3698	0.76 (0.63, 0.93)	3030	10241	1.15 (0.98, 1.36)

[a] Neighborhood Deprivation Quintiles with Q1 being least deprived and Q5 being most deprived

[b] "Healthy" weight defined by having a BMI within [18.5, 24.9]

[c] Obese status defined by having a BMI ≥ 30

[d] Analyses adjusted for age, health insurance status, education, household income, smoking status, and enrollment source; OR=Odds Ratio; CI=Confidence Interval

Stratifications based on the significant likelihood ratio test for interaction by race (p-interaction<0.0001) and sex (p-interaction<0.0001)

Table S2. Associations between Overweight Status and Neighborhood Deprivation, stratified by sex and race, among SCCS at baseline interview, 2002-2009												
	White						Black					
	Males			Females			Males			Females		
Deprivation Index [a]	N, "Healthy" weight [b]	N, Overweight[c]	OR (95% CI) [d]	N, "Healthy" weight [b]	N, Overweight[c]	OR (95% CI) [d]	N, "Healthy" weight [b]	N, Overweight[c]	OR (95% CI) [d]	N, "Healthy" weight [b]	N, Overweight[c]	OR (95% CI) [d]
Q1	465	670	1.0 (ref)	738	562	1.0 (ref)	235	393	1.0 (ref)	212	365	1.0 (ref)
Q2	501	827	1.30 (1.09, 1.54)	883	956	1.29 (1.11, 1.49)	425	564	0.85 (0.68, 1.05)	383	662	1.03 (0.83, 1.27)
Q3	488	734	1.23 (1.04, 1.48)	807	905	1.31 (1.12, 1.53)	579	708	0.87 (0.71, 1.07)	381	759	1.22 (0.98, 1.51)
Q4	559	708	1.08 (0.91, 1.29)	812	946	1.31 (1.12, 1.52)	1188	1350	0.82 (0.68, 0.99)	844	1584	1.15 (0.95, 1.40)
Q5	576	583	1.03 (0.86, 1.24)	498	568	1.33 (1.12, 1.59)	5282	4780	0.73 (0.61, 0.87)	3030	4534	1.01 (0.84, 1.21)

[a] Neighborhood Deprivation Quintiles with Q1 being least deprived and Q5 being most deprived

[b] "Healthy" weight defined by having a BMI within [18.5, 24.9]

[c] Overweight defined by having a BMI within [25, 29.9]

[d] Analyses adjusted for age, health insurance status, education, household income, smoking status, and enrollment source; OR=Odds Ratio; CI=Confidence Interval

Stratifications based on the significant likelihood ratio test for interaction by race (p-interaction<0.0001) and sex (p-interaction<0.0001)

Table S3. Associations between Obese Status and Neighborhood Deprivation in those with insurance, among SCCS at baseline interview, 2002-2009												
	White						Black					
	Males			Females			Males			Females		
Deprivation Index [a]	N, "Healthy" weight [b]	N, Obese [c]	OR (95% CI) [d]	N, "Healthy" weight [b]	N, Obese [c]	OR (95% CI) [d]	N, "Healthy" weight [b]	N, Obese [c]	OR (95% CI) [d]	N, "Healthy" weight [b]	N, Obese [c]	OR (95% CI) [d]
Q1	355	348	1.0 (ref)	578	496	1.0 (ref)	123	201	1.0 (ref)	139	453	1.0 (ref)
Q2	323	510	1.47 (1.18, 1.82)	584	1021	1.53 (1.29, 1.82)	233	332	0.91 (0.67, 1.23)	245	888	1.04 (0.82, 1.33)
Q3	265	507	1.67 (1.33, 2.10)	505	1025	1.67 (1.40, 1.98)	263	391	1.04 (0.77, 1.41)	241	1141	1.42 (1.11, 1.81)
Q4	335	531	1.45 (1.16, 1.82)	513	1158	1.69 (1.42, 2.02)	597	805	1.05 (0.80, 1.39)	503	2563	1.52 (1.22, 1.90)
Q5	254	303	1.17 (0.91, 1.51)	268	622	1.67 (1.36, 2.05)	2401	2136	0.78 (0.60, 1.01)	1705	6519	1.24 (1.01, 1.53)

[a] Neighborhood Deprivation Quintiles with Q1 being least deprived and Q5 being most deprived

[b] "Healthy" weight defined by having a BMI within [18.5, 24.9]

[c] Obese status defined by having a BMI ≥ 30

[d] Analyses adjusted for age, education, household income, smoking status, and enrollment source with reference group those with "healthy" weight; OR=Odds Ratio; CI=Confidence Interval

Stratifications based on the significant likelihood ratio test for interaction by race (p-interaction<0.0001), sex (p-interaction<0.0001), and insurance status in white males (p-interaction=0.009)

Table S4. Associations between Obese Status and Neighborhood Deprivation in those without insurance, among SCCS at baseline interview, 2002-2009												
	White						Black					
	Males			Females			Males			Females		
Deprivation Index [a]	N, "Healthy" weight [b]	N, Obese [c]	OR (95% CI) [d]	N, "Healthy" weight [b]	N, Obese [c]	OR (95% CI) [d]	N, "Healthy" weight [b]	N, Obese [c]	OR (95% CI) [d]	N, "Healthy" weight [b]	N, Obese [c]	OR (95% CI) [d]
Q1	110	105	1.0 (ref)	160	224	1.0 (ref)	112	99	1.0 (ref)	73	242	1.0 (ref)
Q2	178	249	1.62 (1.13, 2.32)	299	603	1.47 (1.13, 1.91)	192	154	0.90 (0.62, 1.31)	138	450	1.04 (0.74, 1.45)
Q3	223	215	1.10 (0.77, 1.58)	302	618	1.73 (1.33, 2.24)	316	254	1.03 (0.73, 1.45)	140	646	1.46 (1.05, 2.04)
Q4	224	250	1.22 (0.85, 1.73)	299	687	1.74 (1.34, 2.26)	591	441	0.92 (0.67, 1.28)	341	1348	1.27 (0.94, 1.71)
Q5	322	211	0.81 (0.57, 1.15)	230	417	1.49 (1.13, 1.96)	2881	1562	0.73 (0.54, 0.98)	1325	3722	1.01 (0.76, 1.34)

[a] Neighborhood Deprivation Quintiles with Q1 being least deprived and Q5 being most deprived

[b] "Healthy" weight defined by having a BMI within [18.5, 24.9]

[c] Obese status defined by having a BMI ≥ 30

[d] Analyses adjusted for age, education, household income, smoking status, and enrollment source with reference group those with "healthy" weight; OR=Odds Ratio; CI=Confidence Interval

Stratifications based on the significant likelihood ratio test for interaction by race (p-interaction<0.0001), sex (p-interaction<0.0001), and insurance status in white males (p-interaction=0.009)

Table S5. Associations between Obese Status and Neighborhood Deprivation in those with household income <$50,000, among SCCS at baseline interview, 2002-2009												
	White						Black					
	Males			Females			Males			Females		
Deprivation Index [a]	N, "Healthy" weight [b]	N, Obese [c]	OR (95% CI) [d]	N, "Healthy" weight [b]	N, Obese [c]	OR (95% CI) [d]	N, "Healthy" weight [b]	N, Obese [c]	OR (95% CI) [d]	N, "Healthy" weight [b]	N, Obese [c]	OR (95% CI) [d]
Q1	239	245	1.0 (ref)	406	530	1.0 (ref)	200	209	1.0 (ref)	156	569	1.0 (ref)
Q2	383	572	1.47 (1.16, 1.87)	687	1419	1.54 (1.30, 1.81)	391	380	0.84 (0.65, 1.09)	336	1216	0.98 (0.79, 1.22)
Q3	407	601	1.40 (1.11, 1.77)	689	1566	1.72 (1.46, 2.03)	557	570	0.98 (0.77, 1.25)	357	1672	1.29 (1.04, 1.61)
Q4	493	682	1.34 (1.06, 1.68)	706	1732	1.81 (1.53, 2.13)	1145	1122	0.92 (0.73, 1.15)	802	3742	1.29 (1.06, 1.57)
Q5	543	459	0.94 (0.74, 1.19)	461	989	1.68 (1.40, 2.01)	5191	3532	0.68 (0.55, 0.84)	2980	9977	1.02 (0.85, 1.24)
[a] Neighborhood Deprivation Quintiles with Q1 being least deprived and Q5 being most deprived												
[b] "Healthy" weight defined by having a BMI within [18.5, 24.9]												
[c] Obese status defined by having a BMI ≥ 30												
[d] Analyses adjusted for age, education, household income, smoking status, and enrollment source with reference group those with "healthy" weight; OR=Odds Ratio; CI=Confidence Interval												
Stratifications based on the significant likelihood ratio test for interaction by race (p-interaction<0.0001), sex (p-interaction<0.0001) , and household income in black females (p-interaction=0.001)												

Table S6. Associations between Obese Status and Neighborhood Deprivation in those with household income ≥$50,000, among SCCS at baseline interview, 2002-2009												
	White						Black					
	Males			Females			Males			Females		
Deprivation Index [a]	N, "Healthy" weight [b]	N, Obese [c]	OR (95% CI) [d]	N, "Healthy" weight [b]	N, Obese [c]	OR (95% CI) [d]	N, "Healthy" weight [b]	N, Obese [c]	OR (95% CI) [d]	N, "Healthy" weight [b]	N, Obese [c]	OR (95% CI) [d]
Q1	226	208	1.0 (ref)	332	190	1.0 (ref)	35	91	1.0 (ref)	56	126	1.0 (ref)
Q2	118	187	1.63 (1.20, 2.21)	196	205	1.57 (1.19, 2.07)	34	106	1.22 (0.69, 2.17)	47	122	1.12 (0.70, 1.80)
Q3	81	121	1.38 (0.97, 1.97)	118	140	1.85 (1.35, 2.54)	22	75	1.44 (0.75, 2.74)	24	115	2.20 (1.26, 3.83)
Q4	66	99	1.39 (0.95, 2.04)	106	113	1.59 (1.14, 2.22)	43	124	1.40 (0.80, 2.44)	42	169	1.83 (1.14, 2.94)
Q5	33	55	1.58 (0.97, 2.56)	37	50	1.85 (1.15, 3.01)	91	166	0.80 (0.49, 1.33)	50	264	2.63 (1.66, 4.15)
[a] Neighborhood Deprivation Quintiles with Q1 being least deprived and Q5 being most deprived												
[b] “Healthy” weight defined by having a BMI within [18.5, 24.9]												
[c] Obese status defined by having a BMI ≥ 30												
[d] Analyses adjusted for age, education, household income, smoking status, and enrollment source with reference group those with “healthy” weight; OR=Odds Ratio; CI=Confidence Interval												
Stratifications based on the significant likelihood ratio test for interaction by race (p-interaction<0.0001), sex (p-interaction<0.0001), and household income in black females (p-interaction=0.001)												
